# Nimodipine Nanoparticles: A Promising Approach for Glaucoma Management

**DOI:** 10.3390/pharmaceutics17111363

**Published:** 2025-10-22

**Authors:** Doaa N. Maria, Sara N. Maria, Monica M. Jablonski, Mohamed Moustafa Ibrahim

**Affiliations:** 1 Department of Ophthalmology, Hamilton Eye Institute, University of Tennessee Health Science Center, Memphis, TN 38163, USA; dmaria@uthsc.edu (D.N.M.); smaria1@uthsc.edu (S.N.M.); mjablonski@uthsc.edu (M.M.J.); 2 Department of Pharmaceutics, Faculty of Pharmacy, Mansoura University, Mansoura 35516, Egypt; 3 Department of Pharmaceutical Sciences, University of Tennessee Health Science Center, Memphis, TN 38163, USA

**Keywords:** nimodipine, carboxymethyl chitosan, low molecular weight chitosan, medium molecular weight chitosan, nanoparticles, transcorneal permeability, cytotoxicity, glaucoma, IOP reduction

## Abstract

**Background/Objectives**: Glaucoma is a multifactorial eye disease that can cause optic nerve damage and irreversible blindness. It is considered a significant public health problem worldwide. Topical intraocular pressure (IOP)-lowering eye preparations are used to prevent or slow further damage. Previously, we demonstrated that nimodipine (NMD), a calcium channel blocker, significantly reduced IOP after a single drop of NMD/HPMC suspension. The current study was designed to develop NMD chitosan nanoparticles (NMD-CS NPs) to improve the NMD IOP-lowering efficacy. **Methods**: NMD-CS NPs were prepared using the spontaneous-emulsification solvent diffusion method. Three different types of chitosan, carboxymethyl CS (CMCS), low molecular weight CS (LCS), and medium molecular weight CS (MCS), were used. Different concentrations of polymers, various stabilizers, and two pHs were used for formulation optimization. NMD-CS NPs were characterized regarding their particle size, polydispersity index (PDI), zeta potential, DSC, FTIR, and encapsulation efficiency. NMD-CS NPs were incorporated into eye drops and characterized in terms of their in vitro release, cytotoxicity, transcorneal permeability, and in vivo efficacy. **Results**: The optimized NMD-CS NPs demonstrate a small particle size with a narrow size distribution and acceptable zeta potential values. DSC and FTIR results confirmed the complete entrapment of NMD inside the NPs. NMD-CS NP eye drops successfully sustained NMD release without any burst effect. These NPs demonstrated a Higuchi non-Fickian diffusion mechanism and 79.41% improved corneal permeability. Cytotoxicity studies revealed that NMD formulations are nontoxic. After a single topical ocular application, NMD-MCS NP eye drops induced a significantly superior effect to Timolol maleate eye drops with regard to the %IOP reduction and duration of action. **Conclusions**: Evaluation results of NMD-CS NP eye drops show their positive effect in a preclinical animal model as a promising glaucoma therapy.

## 1. Introduction

Glaucoma is a multifactorial disease and is considered a leading cause of irreversible blindness worldwide [1]. Trends predict that by 2040, as many as 111.8 million people worldwide will have glaucoma [2]. Many of those patients will be legally blind due to optic nerve (ON) damage associated with glaucoma [3,4]. Several risk factors are known for this disease [5,6,7], with elevated intraocular pressure (IOP) [4,5,8,9,10] and fluctuations in IOP [11,12,13] being the only modifiable risk factors linked to the development and progression of visual field loss [4,5,8,9,10]. Because of the critical role played by IOP regulation in the disease progression, the current standard of care for glaucoma includes treatment with IOP-lowering medications that are delivered topically as eye drops. Various classes of FDA approved antiglaucoma medications have been developed to lower IOP. These classes include carbonic anhydrase inhibitors (e.g., dorzolamide [14]), prostaglandin analogs (e.g., latanoprost [15]), α-adrenergic agonists (e.g., brimonidine [16]), and beta blockers (e.g., timolol [17]). These current antiglaucoma medications are not a cure for the disease. Moreover, they are not universally effective in reducing IOP, and they have multiple side effects [18]. Therefore, the advancement of an effective drug that modulates IOP while minimizing the risk of side effects is still an urgent unmet medical need. Unfortunately, notwithstanding the convenience of instilling eye drops at home, patient adherence is a major challenge, making it essential that topical dosage forms be engineered to mitigate side effects, prolong the IOP-lowering duration, and eliminate IOP fluctuations. To directly address these limitations, previously, we demonstrated that nimodipine (NMD), an L-type voltage-gated calcium channel blocker drug with affinity for the α1 pore of the channel, is considered a new class of antiglaucoma medication. It has the ability to reduce the IOP in a dose-dependent manner in C57BL/6J (B6) mice after a single eye drop application of NMD-hydroxypropyl methyl cellulose (HPMC) suspension (0.1–0.9%). Additionally, our data demonstrated that 0.6% NMD HPMC suspension eye drops induced a significant IOP reduction effect in Dutch belted rabbits [17]. Thus, NMD could serve as a promising IOP-lowering therapy for the management of glaucoma. However, NMD HPMC suspension eye drops only maintain the IOP-lowering ability of the drug for up to 6 h in either mice or rabbits’ eyes. In addition, NMD is a BCS class II drug with very low aqueous solubility [19]. The hydrophobicity of NMD may hinder its ophthalmic use, because it may lower its ocular bioavailability when applied in the form of an aqueous drug suspension. Therefore, a sustained-release ocular formulation of NMD is urgently required to support its application for glaucoma treatment.

To our knowledge, our previously published study was the first to report the IOP-lowering efficacy of NMD as a novel glaucoma therapy. Although, there are multiple research attempts to improve NMD bioavailability for oral, transdermal, or injectable use [17]. These attempts include loading the drug into solid lipid nanoparticles [20], nanocrystals [21], nanoparticles [22], nanoemulsions [23], solid self-nanoemulsifying drug delivery systems [24], and liposomes [25]. Because of the hydrophobic nature of NMD, the development of drug-loaded polymeric nanoparticles is one of the main approaches that would improve its pharmacological response as a promising new glaucoma therapy while promoting a sustained drug release [16,26,27].

Careful polymer selection is the most important factor, which mainly affects the physicochemical properties of the produced polymeric nanoparticles in order to develop an efficient polymeric nanoparticle-based ocular drug delivery system. The use of natural biocompatible polymers, especially polysaccharides, in drug delivery has attracted a lot of interest due to their desirable biodegradable, biocompatible, and hydrophilic properties [28]. Chitosan (CS) is a linear biopolyaminosaccharide, which is prepared under alkaline conditions by deacetylating chitin. It is considered to be the second most abundant natural biopolymer after cellulose [29]. Chitosan has several significant structural and biological characteristics that encourage its wide application in manufacturing different drug delivery systems. Among these characteristics are its ability to dissolve in aqueous systems, its cationic nature, biodegradation, and bioadhesiveness. Chitosan NPs have been widely used for the entrapment of different therapeutic agents as part of the preparation of bioadhesive topical ocular drug delivery systems [30]. Barwal et al. reported the use of CS to prepare brimonidine-loaded CS nanoparticles with improved delivery of the drug to the anterior chamber of the eye and high efficacy as a new ocular sustained-release nanodrug delivery system for glaucoma treatment [31]. Shinde et al. reported the development of nanoparticles from a CS derivative—N-trimethyl CS—that offered a prolonged-release transmucosal ocular delivery of the hydrophobic flurbiprofen in the treatment of bacterial conjunctivitis [32]. Also, Ibrahim et al. reported the improvement of the brimonidine IOP-lowering effect upon incorporation into CS-based nanoparticles compared to commercial eye drops (Alphagan^®^ P) [16]. Chitosan is available in different types, which make it suitable for a wide variety of applications. In the current study, we selected CMCS as an example of water-soluble chitosan that does not require pH control during preparation. Also, LCS and MCS were chosen to study the effect of chitosan molecular weight on the properties of the prepared NPs.

NMD is 3-(2-methoxyethyl)5-propan-2-yl2,6-dimethyl-4-(3-nitrophenyl)-1,4-dihydropyridine-3,5-dicarboxylate with a very low aqueous solubility, 0.067 × 10^−4^ M, and extensive first-pass metabolism. NMD has high lipophilicity and hence easily crosses the blood–brain barrier [17]. To improve the bioavailability of NMD as an IOP-lowering medication, in the current study, we strive to improve its poor bioavailability by incorporating NMD into a bioadhesive chitosan-based polymeric nanoparticulate system. We expect that the inclusion of NMD in a nanoparticulate system made of a hydrophilic bioadhesive polymer like chitosan for topical ocular use could greatly improve its poor bioavailability and its IOP-lowering efficacy. So, in the current study, NMD NPs will be prepared using the emulsification solvent diffusion technique utilizing three different types of chitosan. The prepared NPs will be evaluated using different in vitro, ex vivo, and in vivo tests.

## 2. Materials and Methods

### 2.1. Materials

Nimodipine (NMD) (≥98% purity, molecular weight 418.44 g/mol), low molecular weight chitosan (LCS, MW: 50–190 KDa, degree of deacetylation ≥75%), medium molecular weight chitosan (MCS, MW: 190–310 KDa, degree of deacetylation ≥75%), pluronic F-68 (PF-68, aka, poloxamer 188), polyoxyethylene–polyoxypropylene block copolymer (average molecular weight 8350 Da), polyvinyl alcohol (PVA, MW: 31,000–50,000 Da), sodium phosphate dibasic, potassium chloride, potassium dihydrogen phosphate, sodium bicarbonate, calcium chloride dihydrate, glutathione disulfide, sodium lauryl sulfate (SLS), glacial acetic acid, Triton-X 100, methyl thiazol tetrazolium (MTT), and ethyl acetate were purchased from Sigma-Aldrich (St. Louis, MO, USA). Keratinocyte SFM serum free medium was purchased from Life Technologies Corporation (Grand Island, NY, USA). DMEM/F-12 (Dulbecco’s modified Eagle’s medium/nutrient mixture F-12) was purchased from Mediatech, Inc. (Manassas, VA, USA). Carboxymethyl chitosan (CMCS) was purchased from Santa Cruz Biotechnology (Dallas, TX, USA). Soybean L-α-lecithin (97.1% phosphatidylcholine, 0.3% triglycerides) was purchased from Calbiochem (San Diego, Darmstadt, Germany). Dimethyl sulfoxide (DMSO), sodium chloride, magnesium chloride hexahydrate, dextrose, acetonitrile (HPLC grade), and methanol (HPLC grade) were purchased from Fisher Scientific (Fair Lawn, NJ, USA). Ethyl alcohol was purchased from Decon Labs, Inc. (King of Prussia, PA, USA). A Timolol maleate 0.5% eye drop free sample was received from the eye clinic, Hamilton Eye Institute, University of Tennessee Health Science Center.

### 2.2. Animals

Dutch belted rabbits (4 males and 4 females) were procured from Covance Inc. (Princeton, NJ, USA), weighing 1.8–2.8 Kg, and were aged 16 months. All procedures including rabbits were approved by the Animal Care and Use Review Board of the University of Tennessee Health Science Center (UTHSC) and complied with the Association of Research in Vision and Ophthalmology (ARVO) Statement for the Use of Animals in Ophthalmic and Vision Research in addition to the guidelines for laboratory animal experiments (Institute of Laboratory Animal Resources, Public Health Service Policy on Humane Care and Use of Laboratory Animals).

### 2.3. Nimodipine HPLC Assay

A previously published C18-RP HPLC-UV method was used with minor modifications for the quantification of nimodipine through the current study [33]. An HPLC system (KNAUER, Azura, Germany) equipped with a Supelco Kromasil C18 column (5 μm, 100 Å, 4.0 mm × 300 mm) was used. The mobile phase consisted of a mixture of acetonitrile: methanol: water (55:11:34, *v*/*v*/*v*), which was used for isocratic elution of nimodipine at a flow rate of 0.5 mL/min. The column was kept at 40 °C, and the drug was detected after 18 min at a λ_max_ of 235 nm using a photodiode array detector.

### 2.4. Preparation of NMD Nanoparticles

The spontaneous oil-in-water (O/W) emulsification solvent diffusion method was used for the preparation of NMD-CS NPs with few modifications [34,35]. Table 1 shows the different combinations of excipients that were used for the preparation and optimization of different blank CS NPs. All excipients, parameters, ratios, and combinations of the chemicals shown in Table 1 were selected based on the results of a long preliminary study. Three types of chitosan (CMCS, LCS, and MCS) were used at different concentrations. Also, two types of stabilizers, PF-68 and PVA, were used at 0.5% and 1% w/v, respectively. The effect of lecithin absence on the NP formation was studied. Two different pHs—4.5 and 5.7—were tested during the preparation of both LCS and MCS NPs. In brief, the aqueous phases were prepared by dissolving chitosan in the stabilizer solution PF-68 or PVA using a magnetic stirrer. Deionized water was used for CMCS, while diluted acetic acid solution was used to dissolve LCS and MCS at 2 different pH levels (4.5 and 5.7). The organic phase was prepared by dissolving soybean lecithin in ethyl acetate, and then NMD was dissolved in this organic phase. The organic phase was injected into the magnetically stirred aqueous phase at an injection flow rate of 0.8 mL/min using an infusion pump (Fisher Scientific, Fair Lawn, NJ, USA). The formed emulsion was sonicated (amplitude 30%, 10 min) using a probe sonicator (Ultrasonic homogenizer Model 150 V/T, Biologics, Inc., Cary, NC, USA) in an ice bath. The organic solvent was allowed to evaporate by stirring the emulsion overnight at 200 rpm at room temperature. NPs were collected by ultracentrifugation at 60,000 rpm for 2 h (SORVALL, WX Ultra Series Centrifuge, Thermo Fisher Scientific Inc., Waltham, MA, USA), then washed three times with deionized water and recentrifuged to remove excess emulsifier and unincorporated drug. The final NP pellets were resuspended in trehalose solution (5% *w*/*v*) as a cryoprotectant and lyophilized (Freezone Lyophilizer, Labconco Corporation, Kansas City, MO, USA). NPs used for DSC and FTIR were lyophilized without trehalose. The lyophilized NP powder was kept in a refrigerator (2–8 °C) until further analysis, except for TEM examination, where freshly prepared NPs were used. Blank NPs were prepared using the same procedure, excluding the addition of NMD to the organic phase [34,35].

### 2.5. Optimization of NMD Nanoparticles

For optimization of NMD-NPs, the effects of various formulation variables on the physicochemical properties of NPs were studied, such as the polymer type and concentration, drug/polymer ratio, pH of aqueous phase, stabilizer type, and the use of lecithin. Three different types of chitosan—carboxymethyl chitosan (CMCS), low MW chitosan (LCS), and medium MW chitosan (MCS) at different concentrations—were tested (Table 1). The criteria for selecting the optimum NP formulation for each polymer included the smallest particle size and PDI in combination with the highest zeta potential [35].

### 2.6. Characterization of Optimized NMD Nanoparticles

#### 2.6.1. Average Particle Size, Polydispersity Index (PDI), and Zeta Potential

The average particle size, PDI, and zeta potential of the prepared blank and NMD-NPs were measured after suitable dilution with deionized water using Zetasizer Pro (Malvern Instruments Ltd., Malvern, UK). All measurements were performed at 25 °C. The results of three independent test runs were presented as the mean ± SEM. The data were statistically analyzed using the one-way analysis of variance (ANOVA) test, followed by Tukey’s post hoc test. Statistical calculations were carried out using GraphPad Prism 10 software (GraphPad Software Inc., San Diego, CA, USA) [36].

#### 2.6.2. Transmission Electron Microscopy Examination (TEM)

The shape, surface morphology, and particle size of NMD-NPs were examined using a transmission electron microscope (TEM) (JEM-2000EX II, JEOL, LTD, Tokyo, Japan). The NP suspension (2 µL) was applied to 400-mesh Formvar-coated copper grids (Electron Microscopy Sciences EMS, Hatfield, PA, USA) and kept in a desiccator until complete drying, followed by negative staining with Uranyless EM stain before being examined by TEM (Electron Microscopy Sciences EMS, Hatfield, PA, USA) [37].

#### 2.6.3. Fourier Transform Infrared (FTIR) Spectroscopy Analyses

The FTIR analysis was carried out using a PerkinElmer Spectrum TM 100 FT-IR spectrometer (Shelton, CT, USA) using the Analyst software, Spectrum 6. FTIR spectra of NMD, CS polymers, the physical mixture (PM) of NMD and CS polymers in a 1:1 weight ratio, and the optimized lyophilized NMD-NPs were derived in the scanning range of 650–4000 cm^−1^ [17].

#### 2.6.4. Differential Scanning Calorimetric (DSC) Analyses

The thermograms of NMD, CS polymers, the physical mixture (PM) of NMD and CS polymers in a 1:1 weight ratio, and the optimized freeze-dried NMD-NPs were recorded using a Perkin Elmer Diamond differential scanning calorimeter (DSC) equipped with Pyris software, version 7 (Shelton, CT, USA). Samples were accurately weighed and hermetically sealed in a crimped aluminum pan. The heating rate employed was 20 °C/min, and the samples were scanned in the temperature range of 30–400 °C [17].

#### 2.6.5. Entrapment Efficiency, Drug Loading, and Production Yield Determination

For determination of the % drug encapsulation efficiency, drug loading, and NP yield, 5 mg of lyophilized NMD-NPs was dissolved in 2 mL of DMSO in a 25 mL volumetric measuring flask and brought up to volume with methyl alcohol. The NMD content of the obtained solution was determined by the UV-HPLC method described before [33]. The % entrapment efficiency of NMD (%EE), % drug loading (%DL), and % NP yield (%Y) were assessed three times and calculated using Equations (1)–(3), respectively. Results are presented as the mean ± SEM.

% EE = [weight of entrapped drug/total weight of added drug] × 100 (1)

% DL = [weight of entrapped drug/total dry weight of NPs] × 100 (2)

% Y = [total dry weight of NPs/added (drug + polymer) weight] × 100 (3)

### 2.7. Preparation of NMD-NP Eye Drops

NMD-CMCS, LCS, and MCS NP eye drops were prepared by accurately weighing lyophilized NMD NPs equivalent to 0.3% NMD and homogenously suspending them in PBS pH 7.4. The suspensions were stirred using a magnetic stirrer at 600 rpm for 10 min to ensure a homogeneous NPs distribution. The prepared eye drops were placed in air-tight glass vials and kept at 2–8 °C until used for further studies. The vials were labeled “shake before use” to ensure a homogeneous NP distribution during testing [35]. 

### 2.8. Characterization of NMD-NP Eye Drops

The prepared NMD-NP eye drops were evaluated using the in vitro, ex vivo, and in vivo evaluation tests described below.

#### 2.8.1. In Vitro Release of NMD from NMD-NP Eye Drops and Its Kinetics

The in vitro drug release from NMD-CMCS, LCS, and MCS NP eye drops and the control formulation (NMD aqueous suspension in PBS pH 7.4) was performed using our previously published protocols [16,17]. We used fast micro-equilibrium dialyzers, 1500 µL capacity (Harvard Apparatus Co., Holliston, MA, USA), to which semipermeable regenerated cellulose membranes with molecular weight cut-off 10,000 Da (Harvard Apparatus Co., Holliston, MA, USA) were attached. One hundred microliters of the prepared NMD-NP eye drops were placed in the donor chamber. Meanwhile, the receptor chambers were filled with 1.5 mL warm PBS pH 7.4 containing 0.5% SLS at 35 ± 0.5 °C. SLS was used to improve NMD solubility and help to achieve sink conditions during the release study. The dialyzers were kept in a thermostatically controlled shaker (35 ± 0.5 °C and 100 rpm). At predetermined time intervals ranging from 0.5 to 72 h, the entire medium in the receptor chambers was withdrawn and replaced by 1.5 mL of fresh warmed release medium. All experiments were performed in triplicate, and the concentrations of NMD in the samples were determined by the UV-HPLC method described before [33]. The cumulative percent amount released of NMD was calculated as the mean ± SEM. The release kinetics were studied by fitting the release data using different release kinetics models—zero order, first order, Higuchi, and Korsmeyer–Peppas—to determine the model that best fit with our release data [38,39,40]. The data were analyzed for statistical significance using one-way analysis of variance (ANOVA) followed by Tukey’s post hoc test. Kinetics and statistical analysis were performed using GraphPad Prism 10 software (GraphPad Software, Inc., San Diego, CA, USA).

#### 2.8.2. In Vitro Evaluation of NMD-NP Eye Drops Cell Toxicity

In vitro cytotoxicity of NMD-CMCS, LCS, and MCS NP eye drops was evaluated by MTT assay. The assay was carried out in 96-well plates (Costar 3596, Corning Inc., Corning, NY, USA). One hundred microliters of human corneal limbal epithelial (HCLE) cells were seeded at a concentration of 15 × 10^4^ cell/mL and incubated in a humidified environment at 37 °C in 5% CO_2_ for 24 h in Gibco-Keratinocyte SFM medium containing bovine pituitary extract, epidermal growth factor, and CaCl_2_·2H_2_O. One hundred microliters of the diluted formulations [i.e., 50 μL formulation + 50 μL of medium] were added to each well. Untreated cells and 1% Triton-X 100 served as negative and positive controls for the experiment, respectively. After a 24 h incubation, the formulations as well as the controls were removed, and the plate was washed twice with the culture medium to remove all the traces of the formulations and the controls. One hundred microliters of MTT reagent (1 mg/mL in culture medium without serum) were added to each well, and the plates were incubated at 37 °C in 5% CO_2_ for 4 h. After incubation, the excess MTT was removed, and 100 μL DMSO was added to each well to dissolve the formazan crystals. The plate was shaken for 15 min. The absorbance of the solution was measured at 570 nm by a µ-Quant universal microplate spectrophotometer (Bio Tek Instruments, Inc., Winooski, VT, USA). The absorbances of the treated wells were converted to % cell viability relative to the negative control (the untreated cells). Statistical analysis of the % cell viability data was performed using one-way ANOVA, followed by Tukey’s post hoc test. Each experiment was performed in six replicates, and the results were expressed as the mean ± SEM [35,41].

#### 2.8.3. Ex Vivo Transcorneal Permeability of NMD from NMD-NP Eye Drops

To study the NMD corneal permeability, modified rounded-junction Franz diffusion cells (PermeGear Inc.) were used. Fresh rabbit corneas excised from whole eyes of New Zealand white rabbits that were shipped overnight in Hank’s balanced salt solution over wet ice (Pel-Freez Biologicals LLC, Rogers, AR, USA) and used for that study. Corneas were mounted to the cells with the epithelial side facing up toward the donor chamber containing the formulations—NMD-MCS NP eye drops, and NMD aqueous suspension as a control [42]. One hundred microliters of each formulation were placed in the donor chamber. The receptor chamber was continuously stirred and filled with 5 mL of balanced salt solution enriched with bicarbonate, dextrose, and glutathione, BSS-PLUS (Alcon Laboratories Inc., Fort Worth, TX). With the help of a circulating water bath, the temperature of the cells was maintained at 35 ± 0.5 °C. At predetermined time intervals (1, 2, 3, 4, 5, 6, 7, and 8 h), 500 μL was withdrawn from the receptor chamber and replaced with an equal volume of fresh warmed BSS-PLUS. The drug concentration in withdrawn samples was immediately assayed by the UV-HPLC assay described above. The results were plotted as the cumulative amount permeated (ng) versus time. The flux (J) was calculated from equation (4) by dividing the rate of permeation (dM/dt, slope of the graph) by the surface area of the cornea through which permeation occurred (A), which is equivalent to the orifice area of the Franz diffusion cells (0.636 cm^2^). The permeability coefficient (P) was calculated from equation (5) by dividing the flux by the initial drug concentration in the donor chamber (C_d_). The data were statistically analyzed using *t*-tests. Statistical calculations were carried out using GraphPad Prism 10 software (GraphPad Software Inc., San Diego, CA, USA) [37].

Flux (J) = (dM/dt)/A (4)

Permeability (P) = Flux/C_d_ (5)

#### 2.8.4. In Vivo Evaluation of NMD-NP Eye Drops

The IOP-lowering efficacy of NMD-MCS NP eye drops was evaluated using Dutch belted rabbits. Briefly, 100 μL of 0.3% *w*/*v* NMD-MCS NP eye drops was applied into the inferior conjunctival sac of the right eyes of Dutch belted rabbits (*n* = 5) in two separate applications of 50 μL each, 1 min apart. The left eyes of the rabbits received 100 μL of the control formulation—0.3% *w*/*v* NMD aqueous suspension—in two 50 µL successive doses using our previously published protocol [43]. Another group of Dutch belted rabbits was used to test the %IOP reduction after topical application of a current standard-of-care IOP-lowering medication, Timolol maleate 0.5% eye drops, as a positive control. One drop (~35 µL) of Timolol maleate was applied into the inferior conjunctival sac of both eyes of Dutch belted rabbits (*n* = 3). IOP was measured using a Tono-Pen AVIA Vet (Reichert Technologies, Depew, NY, USA) immediately before treatment (baseline) and at hourly time intervals after application until the IOP returned to its baseline value. For comparison of the 0.3% *w*/*v* NMD-MCS NP eye drops efficacy with that of the two controls—0.3% *w*/*v* NMD aqueous suspension and Timolol maleate 0.5% eye drops—different pharmacodynamic (PD) parameters were calculated. The calculated PD parameters included the time required to reach the maximum reduction in IOP (T_max_); the maximum reduction in IOP value at T_max_ (%IOP reduction); the time required for the IOP to return to the baseline value (end of drug effect, T_end_); and the area under the %IOP reduction-vs.-time curve from 0 h to 10 h (AUC_0–10h_). Results were reported as the mean ± SEM. The calculated PD data were statistically analyzed using one-way ANOVA, followed by Tukey’s post hoc test. Calculation of PD parameters and their statistical analysis were performed using GraphPad Prism 10 software (GraphPad Software Inc., San Diego, CA, USA).

## 3. Results and Discussion

### 3.1. Preparation and Optimization of Different Chitosan Nanoparticles

In the current study, the spontaneous emulsification solvent diffusion method was used for the preparation of different CS NPs. This method was selected because of its ability to produce smaller nanoparticles with a uniform particle size distribution compared to other methods. Ethyl acetate was selected as an organic solvent because it favors the formation of smaller and more uniformly sized nanoparticles as it has the smallest solvent–water exchange ratio among different partially water-miscible solvents [44]. 

For NP optimization, the effects of several formulation variables were studied, including chitosan type and concentration, pH, stabilizer, and lecithin. Three different types of chitosan—CMCS, LCS, and MCS—were studied for NPs’ preparation at different concentrations. The criteria for selecting the optimized NP formulation are based on the smallest particle size and PDI, combined with the highest zeta potential value. Smaller nanoparticles with a narrow size distribution always provide better bioavailability behavior due to their ability to easily penetrate different biological barriers [45]. Specifically, for a topically applied ophthalmic formulation, it is reported that a particle size of less than 200 nm is preferably accepted for passive targeting through biological barriers, including the cornea [37]. Regarding the shelf-life stability profile, with a long time of storage, narrow size distribution NPs might sediment and form a loose precipitate, which can be easily redispersed. However, in the case of NPs with different particle sizes, large particle size NPs might sediment first; then, with time, small NPs will sediment and fill the spaces between the large particle size NPs, resulting in the formation of a hard, compact precipitate, which will be difficult to redisperse. In addition, the shelf-life stability profile is highly affected by the NPs’ zeta potential value. A high zeta potential resulted in higher electric repulsion between particles, which kept them in continuous movement in the aqueous suspension and prevented their aggregation, which improves the shelf-life stability of the formulation [46]. The optimized NP formulation was used for further studies after drug loading.

#### 3.1.1. The Effect of Polymer Type and Concentration

Table 1 shows the different combinations of excipients that were used for the preparation of different NPs, along with their characterizations. The results demonstrated that increasing the polymer concentration, regardless of the type of CS, resulted in an increase in the average particle size and the zeta potential of the formed CS NPs (Table 1). Additionally, the type of chitosan used has a direct effect on the zeta potential of the prepared nanoparticles. The results showed that CMCS NPs possess a negative zeta potential, while LCS NPs and MCS NPs carry positive charges (Table 1).

The results listed in Table 1 demonstrate the effect of the polymer concentration on the particle size and the zeta potential. For CMCS NPs, increasing the polymer concentration from 0.1% (*w*/*v*) to 0.5% *w*/*v* resulted in a significant increase in the mean particle size (*p* = 0.0093) from 127.8 ± 1.8 to 157.2 ± 3.5 nm. This increase in NP size may be attributed to the increased viscosity of the aqueous chitosan solution, which leads to a decreased stirring efficiency during NP formation, increasing the possibility of NP agglomeration [47]. Also, the increased viscosity of the aqueous phase during the emulsification step inversely affected the diffusion of the organic solvent from NPs to the aqueous phase, which decreased the division of larger NPs into smaller ones [35]. Similarly, increasing the CMCS concentration from 0.1% *w*/*v* to 0.5% *w*/*v* significantly increased the zeta potential of the produced CMCS NPs from −5.5 ± 0.7 to −9.8 ± 0.3 mV (*p* = 0.0012).

For LCS, under the same preparation conditions, at pH 4.5, the particle size of LCS NPs significantly increased from 106.5 ± 5.1 nm at 0.1% LCS to 152.0 ± 1.3 nm at 0.3% LCS (*p* < 0.0001). Furthermore, at pH 5.7, the particle size of LCS NPs significantly increased upon increasing the LCS concentration from 0.1% (PS: 130.8 ± 2.6 nm) to 0.3% (PS: 168.0 ± 1.5 nm) (*p* < 0.0001). Previously reported results proved that the CS NP size was dependent on the CS concentration, and the smallest CS NP size was achieved using the lowest CS concentration [48]. Similarly, the results showed that increasing the LCS concentration resulted in a marked increase in the positive zeta potential of the produced NPs (*p* = 0.0201 at pH 4.5 and *p* < 0.0001 at pH 5.7). On the other hand, the results showed that changing the concentration of MCS from 0.1% to 0.2% has no significant effect on the particle size at different pH values (*p* = 0.3849 at pH 4.5 and *p* = 0.8707 at pH 5.7). This non-significant increase in the mean particle size of MCS NPs when the polymer concentration increased from 0.1% to 0.2% may be due to the high viscosity of the aqueous phase observed at both concentrations. Because of the high molecular weight of MCS, both MCS concentrations—0.1% and 0.2%—may have an equal effect on the organic solvent diffusion, which resulted in a non-significant difference in the NP size. In other words, the concentration difference may not be enough to show a significant effect on the NP size. However, the results in Table 1 showed that increasing the MCS concentration significantly increases the positive zeta potential of the prepared NPs (*p* < 0.0001 at pH 4.5 and *p* = 0.0015 at pH 5.7).

In conclusion, depending on the data shown in Table 1, the selected optimized NP formulations that will be used for further investigations include 0.1% *w*/*v* LCS at pH 4.5 using PVA as a stabilizer, 0.2% *w*/*v* MCS at pH 4.5 using PF-68 as a stabilizer, and 0.3% *w*/*v* CMCS with PF-68 as a stabilizer. We selected 0.2% *w*/*v* MCS because the prepared NPs have a significantly higher zeta potential compared to 0.1% *w*/*v* MCS, with no significant difference in the measured particle size.

#### 3.1.2. The Effect of pH

The pH of the formulation medium plays a significant role in the formulation behavior of the chitosan polymer. It is reported that the amino groups on the chitosan chain have a pK_a_ value of ~6.5, which means the polymer is only soluble at pH < 6.5 and carries positive charges due to protonation of its amino groups [49]. To study the effect of the pH on the characteristics of the prepared CS NPs, two different pHs, lower than the chitosan pK_a_ value—pH 4.5 and pH 5.7—were tested.

Data in Table 1 illustrated that, regardless of the type of chitosan and its concentration, increasing the pH from 4.5 to 5.7 resulted in a significant decrease in the zeta potential value (*p* < 0.05) and a significant increase in the particle size (*p* < 0.05) of the corresponding NPs. This may be attributed to the basic nature of chitosan due to the presence of amino groups along its chain. Increasing the pH could result in a decrease in the protonation ability of these CS amino groups, which could produce NPs with a lower zeta potential value that tends to aggregate due to the low repulsion force between them, which in turn could result in a bigger particle size [50]. The results also showed that there is no significant effect of pH on the PDI values of the prepared NPs, regardless of the stabilizer or the polymer concentration (*p* > 0.05). Table 1 showed that all the measured PDI values range between 0.245 and 0.376, which indicated the narrow particle size distribution of the prepared NPs. Finally, we can conclude that pH data support the selection of pH 4.5 for the preparation of both LCS NPs at 0.1% *w*/*v* concentration using PVA as a stabilizer and MCS NPs at 0.2% *w*/*v* concentration using PF-68 as a stabilizer. This is because of their small-sized particles, high zeta potential, and narrow size distribution.

#### 3.1.3. The Effect of Stabilizer

To study the effect of stabilizer on the physicochemical properties of the prepared CS NPs, 0.1% *w*/*v* LCS NPs were prepared using two different stabilizers—1% PVA or 0.5% PF-68—that were pre-selected from a previously conducted preliminary study. Table 1 demonstrates that regardless of the pH of the chitosan solution, there is no significant difference between the physicochemical properties (PS, PDI, and ZP) of the NPs prepared using PVA or PF-68 (*p* > 0.05). This non-significant difference may be due to the equal stabilizing effect of both stabilizers.

#### 3.1.4. The Effect of Lecithin

Data shown in Table 1 illustrate that lecithin is a necessary component for NP formation using LCS. This is because lecithin here plays two important roles. In the first, it acts as the oil phase of the emulsion, which is necessary to dissolve the hydrophobic drug. The second role concerns its nature as a hydrophobic emulsifier, which is required to surround the hydrophobic drug molecules after organic-phase evaporation. In the absence of lecithin, no NPs were formed after the organic-phase evaporation despite the presence of PVA or PF-68 as stabilizers in the aqueous medium; instead, the preparation medium converted to a clear viscous gel. These results are consistent with those we previously reported [16]. In conclusion, the results in Table 1 demonstrated that the presence of lecithin is essential for the formation of NPs.

### 3.2. Characterization of Nimodipine–Loaded Nanoparticles

#### 3.2.1. Particle Size, PDI, and Zeta Potential

**Figure 1:**
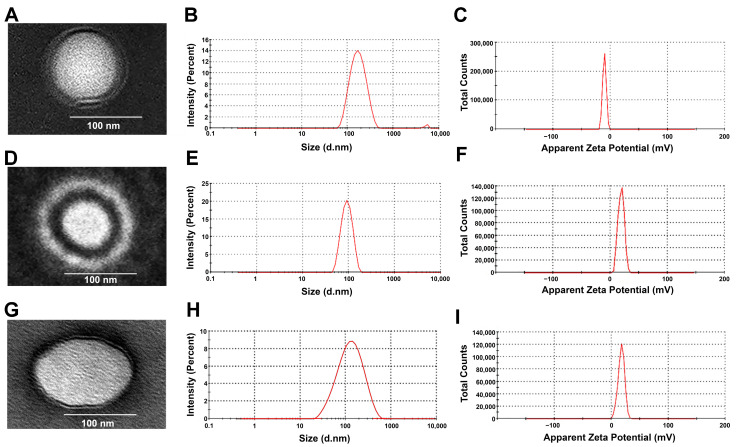
(**A**,**D**,**G**) TEM images of NMD-CMCS NPs, NMD-LCS NPs, and NMD-MCS NPs, respectively, that reveal their spherical outlines with small sizes. (**B**,**E**,**H**) The average particle size of the NMD-CMCS NPs, NMD-LCS NPs, and NMD-MCS NPs, respectively. (**C**,**F**,**I**) Zeta potential of the NMD-CMCS NPs, NMD-LCS NPs, and NMD-MCS NPs, respectively.

Table 2 and Figure 1 illustrate that after encapsulation of NMD into different CS NPs—CMCS NPs, LCS NPs, and MCS NPs—an increase in the hydrodynamic diameter of the NPs was observed. This increase in the particle size was significant in the case of NMD-CMCS NPs (*p* = 0.0037) and non-significant in the case of both LCS NPs and MCS NPs (*p* = 0.3532 and *p* = 0.5480, respectively). Despite the particle size increase, all the prepared drug-loaded NPs are still in the acceptable range of the particle size—< 200 nm—required for passive drug targeting and good in vivo results [16,35]. Data in Table 2 demonstrate that there was no significant change in the PDI value upon drug incorporation for all the prepared CS NPs (*p* > 0.05). Similarly, there was no significant difference between the zeta potential of blank and medicated CS NPs (*p* >0.05), which may be due to the neutral nature of nimodipine molecules, which do not significantly change the ZP of the medicated NPs [51]. In conclusion, the data demonstrated that encapsulation of NMD in different CS NPs did not dramatically change their characteristics, and they are still accepted for passive transport through biological membranes [16].

#### 3.2.2. Encapsulation Efficiency, Drug Loading, and Production Yield

The data presented in Table 2 demonstrate that NMD achieved a high encapsulation efficiency in all the prepared NPs, regardless of the type of chitosan. The results demonstrate that the encapsulation efficiencies of NMD in different CS NPs were in the following order: NMD-CMCS NPs > NMD-LCS NPs > NMD-MCS NPs. Also, the results show that the encapsulation efficiency of NMD in NMD-CMCS NPs was significantly higher than that of both NMD-LCS NPs and NMD-MCS NPs (*p* = 0.0036 and 0.0019, respectively). However, no significant difference was found between NMD-LCS NPs and NMD-MCS NPs (*p* = 0.7656). The reason behind these results may be the enhanced aqueous solubility of NMD in the acidic condition used for the preparation of NMD-LCS NPs and NMD-MCS NPs, which led to the leak of more NMD toward the aqueous phase during the emulsification step rather than staying in the organic phase [52].

Regarding the NP yield, the medicated NPs showed a significant increase in the NP yield compared to the corresponding blank NPs, regardless of the type of CS (overall *p* < 0.001). This significant increase in the medicated NP yield is likely due to the low aqueous solubility of NMD, which led to high drug encapsulation and increased the overall dry weight of the produced NPs. Comparing the NP yield between different polymers indicated that there is a significant difference between all blank NPs (*p* > 0.01) and between all the medicated NPs (*p* > 0.001). Either for blank or medicated NPs, the order of the arrangement of the NP yields is as follows: NMD-LCS NPs > NMD-MCS NPs > NMD-CMCS NPs. The reason for that may be due to the difference in the polymer concentration used between different CS polymers, which is 0.1% for LCS, 0.2% for MCS, and 0.3% for CMCS. This may be due to the increase in the denominator value in equation 3 by increasing the amount of polymer. In addition, our data showed that the % drug loading was 37.22 ± 1.28, 7.52 ± 0.19, and 14.55 ± 0.19 for NMD-CMCS NPs, NMD-LCS NPs, and NMD-MCS NPs, respectively. There is a significant difference in the % drug loading values between CS NPs prepared using different CS polymers (overall *p* < 0.0001). The high % drug loading of NMD-CMCS NPs may be attributed to its high %EE compared to both NMD-LCS NPs and NMD-MCS NPs due to the previously mentioned reasons discussed above.

#### 3.2.3. Surface Morphology of NPs (TEM)

Figure 1A,D,G represent the morphology and shape of NMD-CS NPs. All the prepared NPs were perfectly spherical with a solid, dense core of lecithin surrounded by an evenly distributed coat of CS. TEM images illustrate that all NMD-CS NPs had a particle size in the range of 100–200 nm. These results confirmed the data from the Zetasizer measurement.

#### 3.2.4. Differential Scanning Calorimetric Analysis (DSC)

**Figure 2:**
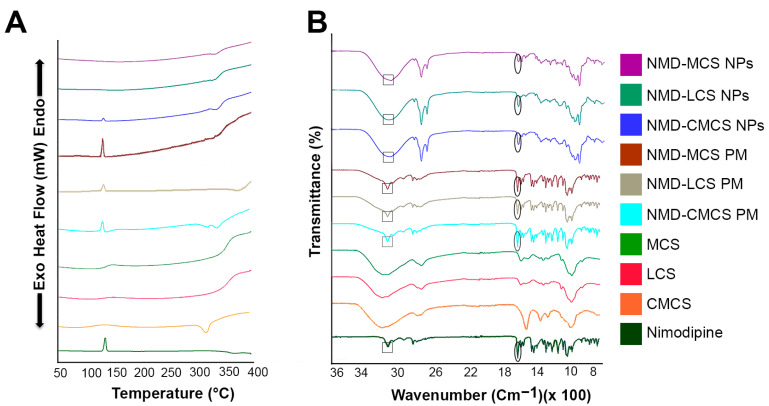
(**A**) Differential scanning calorimetry and (**B**) Fourier transform infrared spectra of NMD, pure chitosan polymers, physical mixtures, and NMD-CS NPs.

DSC thermograms of the pure materials, their physical mixtures (PMs), and the different freeze-dried NPs are illustrated in Figure 2A. The DSC thermal peak data are displayed in Table 3. Pure NMD exhibited a single sharp endothermic peak at 129.69 °C, corresponding to its melting point, which confirmed the crystalline nature of NMD. The DSC pattern of the PMs showed the presence of the characteristic endothermic peak of pure drug with a lower intensity due to the dilution by CS polymers, indicating that the drug retained its crystallinity in the physical mixture. The thermogram of NMD-CMCS NPs showed the presence of the characteristic NMD endothermic peak with a much lower intensity compared to the pure drug or the PM. However, the thermograms of both NMD-LCS NPs and NMD-MCS NPs illustrated a complete disappearance of the characteristic NMD endothermic peak. These data confirmed the complete inclusion of NMD in the amorphous state inside both NMD-LCS NPs and NMD-MCS NPs, and incomplete conversion of NMD from crystalline to an amorphous state in the case of NMD-CMCS NPs. The reason for that may be the difference in the pH of the preparation media during the preparation of these different NPs. In the case of NMD-LCS NPs and NMD-MCS NPs, the acidic pH of the preparation media helped to increase NMD solubility, which led to its incorporation inside NPs in a molecular state that converted to an amorphous form upon evaporation of the solvent. However, during the preparation of NMD-CMCS NPs, the neutral pH of the preparation medium decreased the solubility of NMD and resulted in precipitation of some NMD in the form of nanocrystals, which resulted in the appearance of this very small endothermic peak [52].

#### 3.2.5. Fourier Transform Infrared Analysis (FTIR)

FTIR analysis was carried out to verify the possible physicochemical interactions that could happen between NMD and different CS polymers upon incorporation of NMD into CS NPs. FTIR spectra of the pure NMD are illustrated in the lower panel of Figure 2B over the range of 500–4000 cm^−1^. The FTIR characteristic bands of NMD appeared at 3293 cm^−1^, 1692 cm^−1^, 1520 cm^−1^, and 1196 cm^−1^ due to N–H stretching, carbonyl bond, −NO2 group, and C−O−C stretching, respectively [53]. Chitosan polymers showed characteristic bands at 3361 cm^−1^, 1657 cm^−1^, 1422 cm^−1^, and 1078 cm^−1^ corresponding to O−H stretching, amide band, C−N stretching, and C−O−C stretching, respectively [54]. Figure 2B showed that all NMD characteristic peaks appeared in the FTIR spectra of the three physical mixtures as well as the three prepared CS NPs, either in the same place or with a little shift. These results revealed the absence of any chemical interaction between NMD and CS and confirmed the physical incorporation of NMD inside CS NPs.

### 3.3. In Vitro Release of NMD from Different CS NP Eye Drops and Its Kinetics

**Figure 3:**
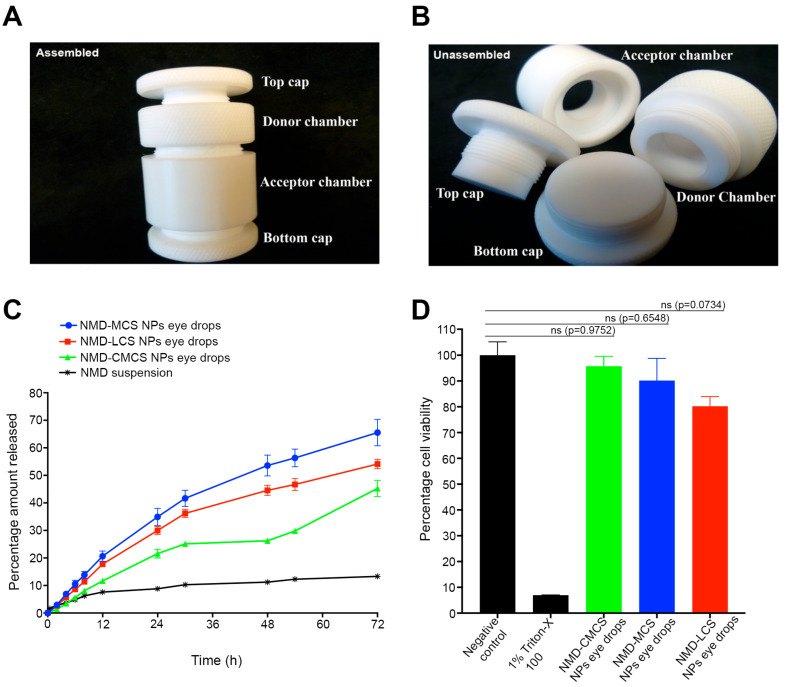
(**A**,**B**) Photographs of the fast micro-equilibrium dialyzer, assembled and unassembled, respectively. (**C**) Percentage cumulative amount of NMD released from different NMD-CS NP eye drops and NMD aqueous suspension (mean ± SEM; *n* = 3). NMD-CS NP eye drops succeeded in improving and sustaining the release of NMD up to 72 h, in contrast to the drug aqueous suspension, which demonstrated only a 13.3 ± 0.2% release of the drug after 72 h. (**D**) Histogram of cytotoxicity of NMD-CMCS NP eye drops, MCS NP eye drops, and LCS NP eye drops using HCLE (mean ± SEM; *n* = 6) demonstrated no cytotoxicity to HCLE cell line after 24 h of incubation.

Figure 3A,B present photographic images of the fast micro-equilibrium dialyzer assembled and unassembled, respectively. Figure 3C demonstrates the in vitro release profiles of NMD from 0.3% NMD-CS NP eye drops, as well as the control formulation (0.3% NMD aqueous suspension). The release profile graphs show that all the NMD-CS NP eye drops demonstrated a sustained drug release behavior without any observed initial burst release.

The percentage cumulative amounts of NMD released from different eye drops after 72 h were 65.5 ± 4.8%, 54.1 ± 1.7%, and 45.3 ± 2.9% for MCS NPs, LCS NPs, and CMCS NP eye drops, respectively. Comparing the release rate of NMD from different NMD-CS NP eye drops yielded the following order: MCS NP eye drops > LCS NP eye drops > CMCS NP eye drops, with no significant difference between MCS NPs and LCS NP eye drops (*p* = 0.0939). This finding may be attributed to two factors. The first factor is the difference in the particle sizes of different NPs. It is known that the smaller the particle size, the higher the surface area available for drug release, and hence the higher the drug release rate [35]. For this reason, CMCS NPs possessed a slower release rate and a lower percentage of the cumulative amount of NMD released because of their larger particle size compared to other NPs (Table 2). The second factor that caused the slower drug release from CMCS NP eye drops was the incomplete incorporation of NMD in amorphous form inside the NPs and the presence of NMD nanocrystals, as confirmed by the DSC data. These nanocrystals slowly dissolve and result in a slower release rate. However, only 13.3 ± 0.2% of NMD was released from the drug aqueous suspension after 72 h, which may be due to the low aqueous solubility of NMD. Comparing the release rates of NMD from its aqueous suspension and NMD-CS NPs revealed that the incorporation of NMD into NPs significantly increased its cumulative amount released (*p* <0.0001), with a remarkable sustained-release behavior.

Release kinetics analysis of NMD in vitro release data from different CS NP eye drops, as well as NMD aqueous suspension, is demonstrated in Table 4. The results illustrated in Table 4 represent the values of the correlation coefficients obtained by fitting NMD in vitro release data to different release kinetics models, including zero, first, and Higuchi kinetics models. After screening of our release data against various release kinetics models, the results demonstrated that, regardless of the type of chitosan, the release kinetics of NMD from different NPs followed a Higuchi release kinetics model (the model with the highest correlation coefficient) [16,55]. Further analysis of the release data using the Korsmeyer–Peppas model revealed that the values of Korsmeyer–Peppas release exponents (*n*) of different NMD-CS NP eye drops were >0.5 and <1.0 (Table 4). Based on these results, we can conclude that the mechanism of NMD release from different CS NP eye drops was not a pure diffusion mechanism; rather, it was an anomalous diffusion mechanism (a mixture of diffusion and polymer swelling and erosion) [35,56]. In contrast, the data showed that the release kinetics of NMD from its aqueous suspension followed zero-order release kinetics, which are only governed by dissolution of the solid drug particles in the release medium (Table 4) [17,55,57]. Because the MCS NP eye drops demonstrated the highest in vitro cumulative amount of NMD released, it was selected for further studies, including ex vivo transcorneal permeability and in vivo evaluation.

### 3.4. In Vitro Cytotoxicity

The HCLE cell line was used to study the potential toxicity of our formulation upon topical application to the eye surface. The results of the in vitro cytotoxicity assay of NMD-CS NP eye drops side by side with both the negative control (untreated cells) and positive control (1% Triton X100) after incubation for 24 h are illustrated in Figure 3D. The data demonstrated that the cell viability of all the tested NMD-CS NP eye drops was significantly different from that of the positive control (*p* < 0.0001), regardless of the type of chitosan used for nanoparticle preparation. Comparing the percentage cell viability values of different NMD-CS NP eye drops with those of the negative control revealed that there was no significant difference (Figure 3D, *p* > 0.05), which indicates the safety of NMD as a drug and the materials used for the preparation of NMD-CS NP eye drops. Typically, 15 min of incubation is sufficient to evaluate the cytotoxicity of conventional topical ocular formulations, which are expected to be eliminated from the eye surface within only 5 min. However, previous studies reported the use of longer incubation times for bioadhesive formulations [58]. Taking into consideration the strong bioadhesion of chitosan polymers that we used for the preparation of NMD-NPs and the expected long contact time of our eye drops, we extended the time frame of our cell viability study to 24 h and demonstrated that no significant reduction in cell viability was observed, which may be attributed to the safety and biocompatibility of NP ingredients [35,56].

### 3.5. Ex Vivo Transcorneal Permeability Study

**Figure 4:**
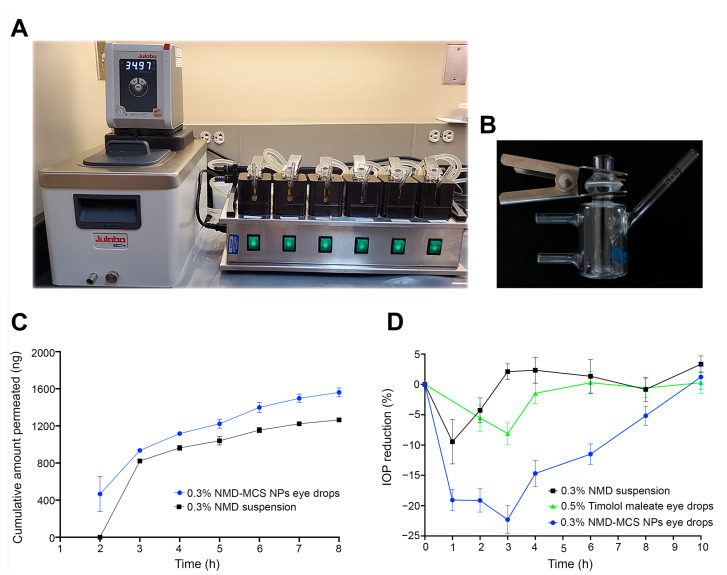
(**A**) Transcorneal permeability Franz diffusion system. (**B**) A single, vertical glass-modified, rounded-junction Franz diffusion cell. (**C**) Ex vivo transcorneal permeability profiles of NMD from 0.3% NMD-MCS NP eye drops and the control, 0.3% NMD aqueous suspension (mean ± SEM, *n* = 5). (**D**) Percentage IOP reduction/time profile after topical application of a single dose of 0.3% NMD-MCS NP eyedrops, 0.3% NMD aqueous suspension, or Timolol maleate 0.5% eye drops (mean ± SEM, *n* = 3–5).

NMD-MCS NP eye drops were selected for ex vivo transcorneal permeability evaluation because it demonstrated the highest in vitro cumulative drug release. To determine the transcorneal permeability of NMD from 0.3% NMD-MCS NP eye drops, as well as from the control (0.3% NMD aqueous suspension), fresh rabbit corneas were mounted on round-junction Franz diffusion cells (Figure 4A,B). To keep the cornea alive during the experiment, BSS-PLUS irrigating solution was used as a receptor medium. NMD is a BCS class II drug that has intrinsic high permeability, which is unfortunately hindered by its poor aqueous solubility [41]. The data shown in both Figure 4C and Table 5 demonstrate that NMD-MCS NP eye drops significantly improve the permeability of NMD by a factor of 79.41% increase in the permeation rates (dM/dt), flux values (J), or permeability coefficients (P) compared to the control (*p* = 0.0078). This improvement in NMD permeability upon incorporation into the CS NPs may be attributed to the small particle size of NMD-MCS NPs, which allows for improved passive transport through corneal tissues [37].

### 3.6. In Vivo Evaluation

NMD-MCS NP eye drops were selected for in vivo evaluation because it demonstrated the highest in vitro cumulative amount of drug released. Dutch belted rabbits were used to determine the IOP-lowering efficiency of 0.3% NMD-MCS NP eye drops and compare it to the control formulation, 0.3% NMD aqueous suspension. These rabbits were selected for this study because they spontaneously develop elevated IOP post-puberty and are widely used as an animal model to test the IOP-lowering effect of different medications [59,60].

Data illustrated in Figure 4D and Table 6 demonstrate that after topical ocular application, 0.3% NMD-MCS NP eye drops induced an IOP reduction of 23.36 ± 1.76% that returned to baseline after 9.60 ± 0.51 h post-dosing. However, the control formulation, 0.3% NMD suspension, induced an IOP reduction of 10.56 ± 3.54% that returned to baseline at 2.20 ± 0.58 h post-dosing. Also, Timolol maleate 0.5% eye drops induced an IOP reduction of 8.1 ± 1.7% that returned to baseline after 4.4 ± 0.6 h post-dosing. In addition, the time of maximum response after topical application of 0.3% NMD-MCS NP eye drops, 0.3% NMD suspension, and Timolol maleate 0.5% eye drops was 2.60 ± 0.43 h, 1.25 ± 0.25 h, and 2.5 ± 0.2 h, respectively (Table 6). As illustrated in Figure 4D, the area under the % IOP reduction-versus-time curve was 118.04 ± 11.08%. h, 38.00 ± 7.43%. h, and 30.0 ± 7.1%. h for 0.3% NMD-MCS NP eye drops, 0.3% NMD suspension, and Timolol maleate 0.5% eye drops, respectively. Statistical analysis of PD parameters demonstrated that there were significant differences between the 0.3% NMD-MCS NP eye drops and Timolol maleate 0.5% eye drops in IOP value at T_max_ (*p* = 0.0008), ∆IOP (*p* = 0.006), % IOP reduction (*p* = 0.0016), T_end_ (*p* < 0.0001), and AUC_0–10h_ (*p* ≤ 0.0001). In addition, there were significant differences between the 0.3% NMD-MCS NP eye drops and 0.3% NMD aqueous suspension in IOP value at T_max_ (*p* = 0.026), ∆IOP (*p* = 0.0186), % IOP reduction (*p* = 0.008), T_end_ (*p* < 0.0001), and AUC_0–10h_ (*p* ≤ 0.0001). These data demonstrated that 0.3% NMD-MCS NP eye drops had an IOP-lowering efficacy superior to both Timolol maleate 0.5% eye drops and 0.3% NMD aqueous suspension regarding both the magnitude (i.e., % IOP reduction) and the duration of action (i.e., T_end_ and AUC_0–10h_). In addition, these results illustrated that the incorporation of NMD into MCS NPs significantly increased its IOP-lowering effect regarding both the magnitude and the duration of action. This superiority in the IOP-lowering effect of NMD-MCS NP eye drops could be attributed to two reasons. The first reason is the strong bioadhesive characteristics of chitosan NPs, which enable the formulation to remain adhered to the eye surface and act as a drug reservoir that continuously releases the drug for a longer period [61]. This improved corneal contact time allowed more drug to penetrate the cornea, resulting in a superior IOP-lowering response compared to the control formulations that drained from the eye surface to the systemic circulation through the nasolacrimal duct within a few minutes after application [55]. The second reason for this enhanced efficacy of NMD-MCS NP eye drops is the improved corneal permeability of the NPs as discussed before [37]. From these data, we can conclude that 0.3% NMD-MCS NP eye drops could be considered an effective IOP-lowering medication, and we support its potential use as a promising glaucoma therapy.

## 4. Conclusions

Glaucoma is the leading cause of irreversible blindness worldwide. Preservation of IOP within physiologically normal limits is a very important strategy in the management of glaucoma and the prevention or slowing down of disease progression. Thus, a reduction in elevated IOP is considered the first-line therapeutic option in glaucoma management. In the current work, we succeeded in developing and evaluating NMD-loaded CS NP eye drops as a promising glaucoma therapy. These drug-loaded CS NPs were prepared using a spontaneous-emulsification solvent diffusion technique. The optimized NMD-CS NPs have small particle sizes with a narrow particle size distribution, an acceptable zeta potential, and a spherical outline. DSC analyses illustrated the absence of NMD characteristic peak(s) in NMD-CS NPs compared to the pure drug and the physical mixture, which confirms the packing of NMD inside the NPs. FTIR analysis proved the absence of chemical interaction between NMD and CS during NP formation. In vitro release data of NMD-CS NP eye drops demonstrated a sustained-release behavior that is free from any initial burst release. Release kinetics analysis of NMD from the eye drops revealed an anomalous diffusion mechanism. Because the MCS NP eye drops demonstrated the highest in vitro cumulative amount of NMD released, it was selected for both ex vivo transcorneal permeability and in vivo evaluation. The in vitro cytotoxicity studies on the HCLE cell line proved that all the prepared NMD-CS NP eye drops are nontoxic. Furthermore, transcorneal permeability results demonstrated that NMD-MCS NP eye drops significantly improve the permeability of NMD compared to NMD aqueous suspension. In vivo evaluation of NMD-MCS NP eye drops using Dutch belted rabbits showed a great improvement in NMD IOP-lowering response after incorporation into CS NPs compared to its aqueous suspension and Timolol maleate 0.5% eye drops. Finally, we can conclude that, because of the ability of NMD-CS NP eye drops to extend NMD release, and extend its IOP-reducing duration of action in a glaucoma animal model, they could be considered a promising drug delivery system for the management of glaucoma.

## Figures and Tables

**Table 1:** Composition and physicochemical properties of blank NPs using three different CS polymers ^a^.

Type of Chitosan	pH	Concentration (% *w*/*v*)				Physicochemical Properties		
		Chitosan	PVA ^b^	PF-68 ^c^	Lecithin	PS (nm) ^d^	PDI ^e^	ZP (mV) ^f^
CMCS	-	0.1	-	0.5	1	127.8 ± 1.8	0.303 ± 0.01	−5.5 ± 0.7
CMCS *	-	**0.3**	-	**0.5**	**1**	**141.1 ± 6.5**	**0.294 ± 0.01**	**−9.5 ± 0.3**
CMCS	-	0.5	-	0.5	1	157.2 ± 3.5	0.320 ± 0.01	−9.8 ± 0.3
LCS	4.5	0.1	1	-	-	No NPs were formed (clear viscous gel)		
LCS *	**4.5**	**0.1**	**1**	-	**1**	**101.9 ± 0.3**	**0.258 ± 0.01**	**18.9 ± 1.1**
LCS	5.7	0.1	1	-	1	123.3 ± 1.4	0.245 ± 0.01	14.0 ± 0.6
LCS	4.5	0.1	-	0.5	1	106.5 ± 5.1	0.314 ± 0.02	16.3 ± 0.9
LCS	5.7	0.1	-	0.5	1	130.8 ± 2.6	0.261 ± 0.01	15.0 ±1.2
LCS	4.5	0.3	-	0.5	-	No NPs were formed (clear viscous gel)		
LCS	4.5	0.3	-	0.5	1	152.0 ±1.3	0.332 ± 0.03	27.2 ± 1.1
LCS	5.7	0.3	-	0.5	1	168.0 ± 1.5	0.376 ± 0.00	21.4 ± 0.5
MCS	4.5	0.1	-	0.5	1	124.6 ± 4.4	0.273 ± 0.00	11.2 ± 0.6
MCS	5.7	0.1	-	0.5	1	128.4 ± 0.2	0.278 ± 0.01	10.04 ± 0.51
MCS *	**4.5**	**0.2**	-	**0.5**	**1**	**130.0 ± 0.3**	**0.284 ± 0.00**	**22.9 ± 0.4**
MCS	5.7	0.2	-	0.5	1	140.4 ± 8.9	0.324 ± 0.04	15.2 ± 0.8

All parameters, ratios, and combinations of the chemicals shown in the table were selected based on the results of a preliminary study. ^a^ Data are expressed as mean ± SEM; *n* = 3. ^b^ PVA: polyvinyl alcohol. ^c^ PF-68: pluronic F-68. ^d^ PS: particle size. ^e^ PDI: polydispersity index. ^f^ ZP: zeta potential. * The selected optimized formulation.

**Table 2:** Physicochemical properties of blank and NMD-NPs prepared with different types of chitosan.

Formulation	Evaluation (Mean ± SEM), *n* = 3					
	Particle Size (nm)	PDI	Zeta Potential (mV)	EE (%)	%DL	%Y
Blank CMCS NPs	141.1 ± 6.5	0.294 ± 0.01	−9.5 ± 0.3	-	-	32.27 ± 1.16
NMD-CMCS NPs	160.2 ± 1.2	0.255 ± 0.02	−10.7 ± 0.2	79.91 ± 2.74	37.22 ± 1.28	40.52 ± 0.79
Blank LCS NPs	101.9 ± 0.3	0.258 ± 0.01	18.9 ± 1.1	-	-	57.56 ± 0.74
NMD-LCS NPs	109.6 ± 1.9	0.275 ± 0.02	18.4 ± 0.3	65.08 ± 1.64	7.52 ± 0.19	72.09 ± 1.33
Blank MCS NPs	130.0 ± 0.3	0.284 ± 0.00	15.2 ± 0.8	-	-	50.95 ± 1.01
NMD-MCS NPs	136.8 ± 0.8	0.233 ± 0.01	15.9 ± 0.7	63.17 ± 0.82	14.55 ± 0.19	62.25 ± 1.19

%EE is the percentage encapsulation efficiency. %DL is the percentage drug loading. %Y is the percentage of NP yield.

**Table 3:** DSC thermal peak data of NMD, CS polymers, physical mixtures (1:1 ratio) (PM), and freeze-dried NPs.

Sample	T_m_ (°C)	T_onset_ (°C)	Peak Height	Area	Delta H (Enthalpy Change) J/g
NMD	129.69	126.59	19.99	410.59	91.86
NMD-CMCS PM	120.46	116.91	6.71	156.27	52.09
NMD-LCS PM	124.56	121.10	4.53	108.95	40.35
NMD-MCS PM	128.83	125.81	9.25	185.96	51.65
NMD-CMCS NPs	125.82	123.55	1.79	28.98	7.83
NMD-LCS NPs	Complete absence of NMD peak				
NMD-MCS NPs	Complete absence of NMD peak				

**Table 4:** NMD in vitro release kinetics from different NMD-CS NP eye drops compared to NMD aqueous suspension as a control.

Formulation	Coefficient of Determination (R^2^)			Korsmeyer–Peppas		Drug Transport Mechanism	Release Mechanism
	Zero	First	Higuchi	r^2^	*n*		
NMD aqueous suspension	**0.985 ± 0.004**	0.903 ± 0.017	0.976 ± 0.005	0.982 ± 0.006	0.435 ± 0.028	xxx	Dissolution
NMD-CMCS NP eye drops	0.914 ± 0.038	0.711 ± 0.046	**0.948 ± 0.015**	0.959 ± 0.018	0.911 ± 0.031	Non-Fickian	Anomalous diffusion
NMD-LCS NP eye drops	0.941 ± 0.013	0.745 ± 0.008	**0.991 ± 0.005**	0.976 ± 0.004	0.826 ± 0.026	Non-Fickian	Anomalous diffusion
NMD-MCS NP eye drops	0.955 ± 0.002	0.729 ± 0.018	**0.997 ± 0.000**	0.976 ± 0.006	0.859 ± 0.056	Non-Fickian	Anomalous diffusion

**Table 5:** Ex vivo transcorneal permeability parameters of NMD from NMD-MCS NP eye drops and the control ^a^.

Formulation	Rate of Permeation (dM/dt) (ng/h)	Flux (ng/cm^2^.h)	Permeability Coefficient (P) × 10^−4^ (cm/h)	% of Permeability Improvement
NMD aqueous suspension	62.20 ± 9.87	97.80 ± 15.52	40.75 ± 6.47	---
NMD-MCS NP eye drops	111.47 ± 5.67	175.27 ± 8.92	73.03 ± 3.72	79.41

^a^ Data are expressed as mean ± SEM; *n* = 5. The *p*-value that represents the outcome of the unpaired *t*-test is 0.0078.

**Table 6:** Pharmacodynamic parameters after topical application of a single dose of 0.3% NMD-MCS NP eye drops and the two controls, 0.3% NMD suspension and 0.5% Timolol maleate 0.5% eye drops ^a^.

Pharmacodynamic Parameters	Formulations		
	0.3% NMD-MCS NP Eye Drops	0.3% NMD Aqueous Suspension	0.5% Timolol Maleate Eye Drops
Baseline IOP ^b^	18.80 ± 0.58	18.8 ± 0.58	19.8 ± 0.7
IOP at T_max_ ^c^	14.40 ± 0.51	16.80 ± 0.80	18.2 ± 0.3
Change in IOP at T_max_(∆IOP)	−4.40 ± 0.40	−2.00 ± 0.71	−1.7 ± 0.4
% IOP Reduction at T_max_	−23.36 ± 1.76	−10.56 ± 3.54	−8.1 ± 1.7
T_max_ (h)	2.60 ± 0.43	1.25 ± 0.25	2.5 ± 0.2
T_end_ (h) ^d^	9.60 ± 0.40	2.2 ± 0.58	4.4 ± 0.6
AUC_0–10h_ (%. h) ^e^	118.04 ± 11.08	38.00 ± 7.43	30.0 ± 7.1

^a^ Data are expressed as mean ± SEM; *n* = 3–5. ^b^ IOP: intraocular pressure. ^c^ T_max_ (h): time to maximum response in hours. ^d^ T_end_ (h): time to end of response in hours. ^e^ AUC_0–10h_ (%. h): area under % IOP reduction-versus-time curve from 0 h to 10 h.

## Data Availability

The raw data supporting the conclusions of this article will be made available by the authors on request.

## Abbreviations

The following abbreviations are used in this manuscript:

NMD	Nimodipine
MCS	Medium-molecular-weight chitosan
LCS	Low-molecular-weight chitosan
CMCS	Carboxymethyl chitosan
IOP	Intraocular pressure
NPs	Nanoparticles
